# N-acetylcysteine for the prevention of stricture after circumferential endoscopic submucosal dissection of the esophagus: a randomized trial in a porcine model

**DOI:** 10.1186/1755-1536-5-8

**Published:** 2012-05-28

**Authors:** Maximilien Barret, Frédéric Batteux, Frédéric Beuvon, Luigi Mangialavori, Ariane Chryssostalis, Carlos Pratico, Stanislas Chaussade, Frédéric Prat

**Affiliations:** 1 Department of Gastroenterology, Cochin Hospital, 27, rue du Faubourg St Jacques, Paris, 75014, France; 2 Department of Immunology and EA 1833, Cochin Hospital, 27, rue du Faubourg St Jacques, Paris, 75014, France; 3 Department of Pathology, Cochin Hospital, 27, rue du Faubourg St Jacques, Paris, 75014, France

**Keywords:** Endoscopic submucosal dissection (ESD), Esophageal stricture, Esophageal fibrosis, Barrett’s esophagus, Early esophageal adenocarcinoma

## Abstract

**Background:**

Circumferential endoscopic submucosal dissection (CESD) of the esophagus would allow for both the eradication of Barrett’s esophagus and its related complications, such as advanced neoplasia. However, such procedures generally induce inflammatory repair resulting in a fibrotic stricture. N-acetylcysteine (NAC) is an antioxidant that has shown some efficacy against pulmonary and hepatic fibrosis. The aim of our study was to evaluate the benefit of NAC in the prevention of esophageal cicatricial stricture after CESD in a swine model.

**Animals and methods:**

Two groups of six pigs each were subjected to general anesthesia and CESD: after randomization, a first group received an oral NAC treatment regimen of 100 mg/kg/day, initiated one week before the procedure, whereas a second group was followed without any prophylactic treatment. Follow-up endoscopies took place seven, fourteen, twenty-one, and twenty-eight days after CESD. Necropsy, histological assessment of esophageal inflammation, and fibrosis were performed on day 28.

**Results:**

The median esophageal lumen diameter on day 21 (main judgment criterion) was 4 mm (range 2 to 5) in group 1 and 3 mm (range 1 to 7) in group 2 (*P* = 0.95). No significant difference was observed between the two groups regarding clinical evaluation (time before onset of clinically significant esophageal obstruction), number of dilations, esophageal inflammation and fibrosis, or oxidative stress damage on immunohistochemistry.

**Conclusions:**

Despite its antioxidant effect, systemic administration of NAC did not show significant benefit on esophageal fibrosis in our animal model of esophageal wound healing within the experimental conditions of this study. Since the administered doses were relatively high, it seems unlikely that NAC might be a valuable option for the prevention of post-endoscopic esophageal stricture.

## Background

Endoscopic resection of Barrett’s esophagus with a high-grade dysplasia or early esophageal cancer has become an acceptable therapeutic option [1-3], alongside surgical esophagectomy. Endoscopic mucosal resection is easily feasible but leads to a piecemeal, non-carcinologic resection of neoplastic tissue, whereas endoscopic submucosal dissection (ESD) is more carcinologically efficient, although more technically challenging. Nonetheless, both techniques result in cicatricial fibroinflammatory esophageal strictures when 75% or more of the mucosal circumference is removed [4,5]. These strictures may require numerous endoscopic dilations, jeopardizing the patients’ quality of life and risking esophageal perforation. This is believed to result from local inflammatory reactions, as well as subsequent cicatricial fibrosis [4,6-8]. Furthermore, pathological wound healing and fibrogenesis in the esophagus has been linked to the production of reactive oxygen species [9].

We developed an endoscopic submucosal dissection technique in a swine model, allowing for quick and safe *en bloc* circumferential resection of the esophageal mucosa [10]. A similar technique has been described since [11]. This method consists of a classical endoscopic submucosal dissection and blunt mechanical dissection using an endoscopic mucosectomy cap. It allows for *en bloc* resection of mucosal and submucosal cylinders up to 15 centimeters, without taking longer or causing more immediate complications than endoscopic mucosal resection alone. However, tight oesophageal strictures always develop in this model between the eighth and the fifteenth postoperative day.

N-acetylcysteine (NAC) is an antioxidant molecule with antifibrotic properties [12,13], mainly related to the inhibition of transforming growth factor-beta (TGF-β) signaling [14]. It has an anti-inflammatory effect caused by downregulating TNF-α, IL-6, and IL-8 synthesis [15]. Furthermore, it is a cheap, readily available, and a safe molecule. NAC has shown efficiency in pulmonary fibrosis [16] and is of possible interest in cirrhotic [17], nonalcoholic steatohepatitis [18] liver fibrosis, and improved wound healing of tympanic membranes [19]. In the esophagus, anastomotic wound healing improved with NAC administration [20].

Therefore, we conducted a controlled trial to assess the interest of NAC in the prevention of esophageal strictures, following circumferential ESD in our swine model.

## Results

**Figure 1:**
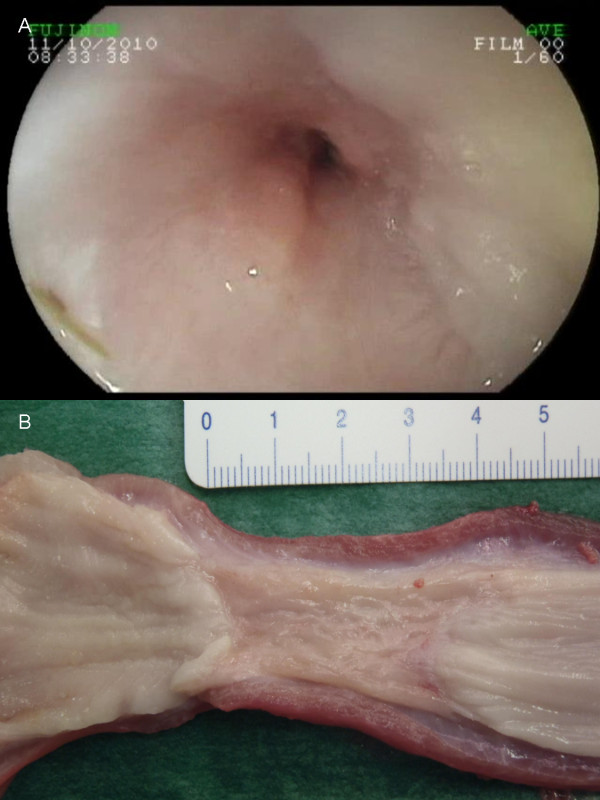
**Esophageal stricture following circumferential endoscopic submucosal dissection.** Endoscopic view (**A**) and gross morphology (**B**).

**Figure 2:**
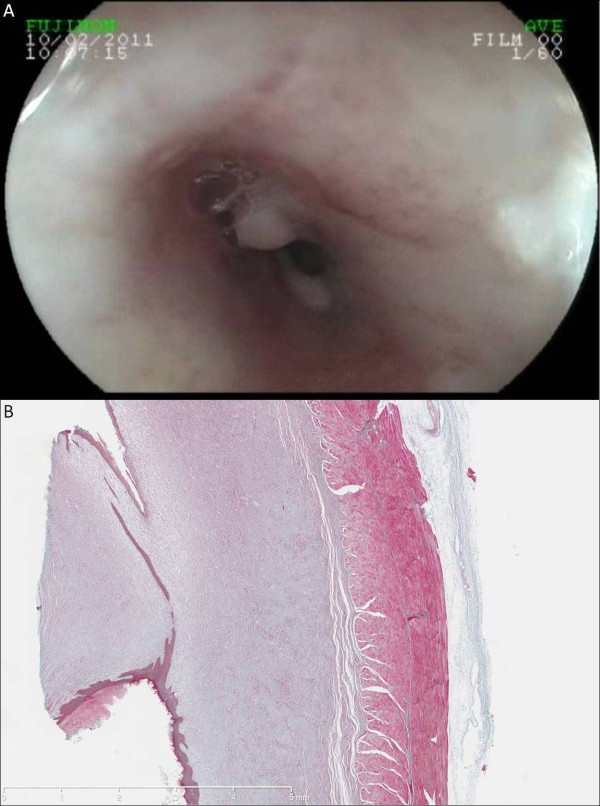
Esophageal stricture following circumferential endoscopic submucosal dissection, endoscopic view (A) and microscopic view (B) (Masson’s trichrome, original magnified x12.5) showing the corresponding histological feature.

The median esophageal diameter on the 21^st^ postoperative day was 4 mm (range 2 to 5) in group 1 (treated with NAC) and 3 mm (range 1 to 7) in group 2 (control) (*P* = 0.95) (Figure 1 and 2).

In group 1, none of the six animals exhibited abnormal behavior, but five out of six (83%) were unable to ingest a regular diet after fourteen days because of regurgitations. In group 2, one pig out of five (20%) was found to be asthenic, and four out of five (80%) were unable to eat because of their esophageal stricture. Overall number of dilation was 13 (2.2 per pig) in group 1 and 12 (2 per pig) in group 2 on day 21 (*P* = NS).

At necropsy, mediastinal periesophageal lymph nodes with evidence for mediastinal inflammation, but no other feature of mediastinitis, were diagnosed in one pig in each group, probably as a result of repeated dilations. In Group 2, two animals experienced an esophageal perforation related to the endoscopic procedure: one was successfully treated with endoscopic clips and antibiotherapy and the other required euthanasia. One of the pigs from Group 2 died of mediastinitis following esophageal dilation on day 22. The number of esophageal dilations and complications recorded during follow-up for each animal are presented in Table 1.

**Table 1:** Number of esophageal dilations and occurrence of complications

**Animal number**	**Number of dilations**	**Early complication**	**Late complication**	**Other findings**
1**	2	-	-	
2*	3	-	-	
3**	0	Post-endoscopic esophageal perforation, requiring euthanasia		
4*	3	-	-	
5**	3	-	-	
6*	1	-	-	
7**	3	-	Mediastinitis on day 22, following the third dilation	
8*	2	-	-	
9**	2	Post-endoscopic esophageal perforation, successfully treated	-	
10*	2	-	-	
11**	2	-	-	Mediastinal inflammation^+^ at necropsy
12*	2	-	-	Mediastinal inflammation^+^ at necropsy

*Group 1, treated by N-acetylcysteine; **group 2, control group; ^+^periesophageal lymphadenopathies without actual mediastinitis.

**Figure 3:**
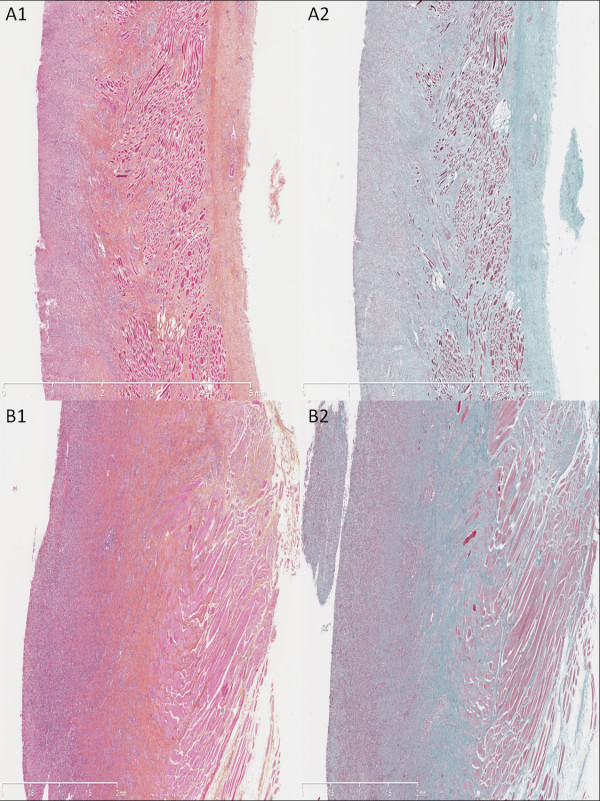
**Histological analysis of the resected esophagus with predominant inflammatory pattern.** Absence of neoepithelium, granulation tissue with an important inflammatory cell infiltrate of the submucosal layer; submucosal fibrosis is highlighted by Masson’s trichrome staining. (**A1**) Untreated swine, hematoxylin-eosin-safran (HES), original magnified x12.5. (**A2**) Untreated swine, Masson’s trichrome, original magnified x12.5. (**B1**) Treated by N-acetylcysteine (NAC), HES, original magnified x25. (**B2**) Treated by NAC, Masson’s trichrome, original magnified x25.

**Figure 4:**
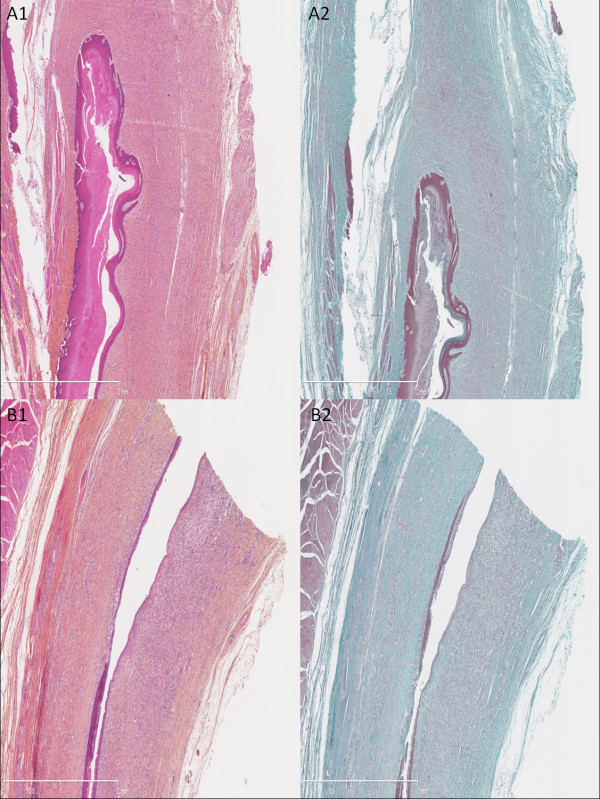
**Histological analysis of the resected esophagus with predominant fibrotic pattern.** Presence of a neoepithelium, as a single layer of immature epithelial cells, a thin granulation tissue with moderate cell density, and important thickness of cicatricial fibrosis infiltrating the remaining submucosal and the muscular layer. (**A1**) Untreated swine, hematoxylin-eosin-safran (HES), original magnified x25. (**A2**) Untreated swine, Masson’s trichrome, original magnified x25. (**B1**) Treated by N-acetylcysteine (NAC), HES, original magnified x25. (**B2**) Treated by NAC, Masson’s trichrome, original magnified x25.

**Figure 5:**
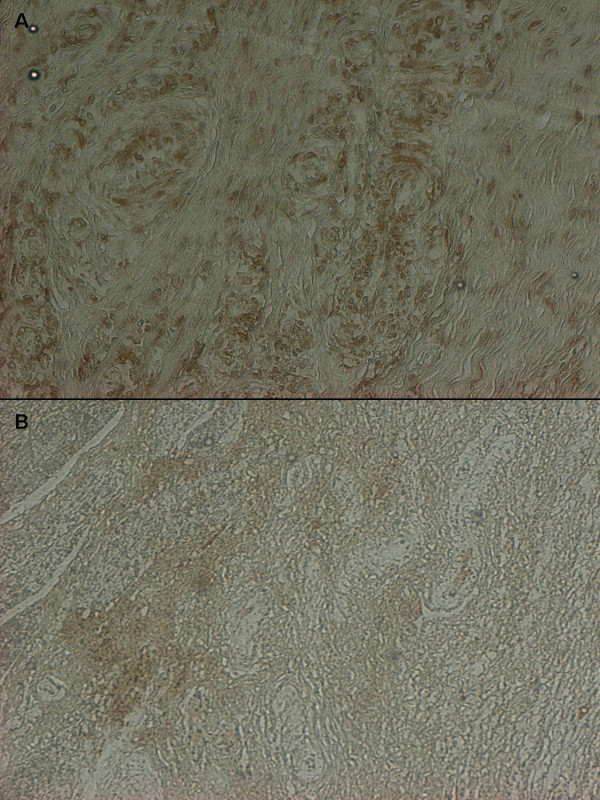
**Immunohistochemical staining with 8-hydroxy-2’ deoxyguanosine antibody.** Strong signal predominant in perivascular region in control (**A**) and almost absent signal in treated swine (**B.**)

Histology revealed two different patterns that were found indistinctively in both groups. In the first one (Figure 3), the superficial layer remained ulcerated; we observed a dense granulation tissue with a major inflammatory cell infiltrate and high cell density with predominance of polynuclear leukocytes in the remaining submucosa; we also observed myofibroblasts in the submucosal and muscular layers, surrounded by fibrosis (Figure 3, A2 and B2). Conversely, we observed a neoepithelium in the second pattern (Figure 4), characterized by a single layer of immature epithelial cells. Fibrosis (Figure 4, A2 and B2) was found underneath the neoepithelium, around myofibroblasts and fibroblasts, filling the submucosal layer and dissecting muscular fibers; associated with moderate, lymphoplasmocytic inflammatory cell infiltrate, and granulation tissue (Figure 4, A and B). These two patterns can be hypothesized as corresponding to the early and late phases of the same inflammatory and fibrotic cicatricial process. Median fibrosis thickness was 1740 μm (range 1030 to 2570) in group 1 vs 1915 μm (range 1710 to 2860) in group 2, *P* = NS; median thickness of the granulation tissue was 1000 μm (range 925 to 1100) in group 1 vs 843 μm (range 868 to 2440) in group 2, *P* = NS. The granulation tissue presented with features of acute inflammation in 30% of cases in group 1 vs 25% in group 2. Immunohistochemistry staining with 8-hydroxy-2’ deoxyguanosine (8-OH DG) antibody showed a more intense signal in the esophageal wall of control animals as compared to NAC-treated animals (Figure 5). This finding was consistently observed on all slides. However, signal measurements did not show a statistically significant difference between the groups: median: 148 (range 122 to 169) in group 1 vs 136 (range 90 to 168) in group 2, *P* = 0.54.

## Discussion

This study is the first one to evaluate, on a swine model, the systemic administration of an antioxidant molecule in the prevention of esophageal strictures.

Nonetheless, this work did not demonstrate any clinical, endoscopical, or histological benefit of NAC to prevent oesophageal stenosis after circumferential ESD. A first hypothesis is an insufficient oral intake of NAC in regurgitating animals, despite repeated esophageal dilations. However, if solid food intake is impaired by esophageal stricture, liquid intake is almost always possible. Moreover, food intake remained normal during the first 10 days, the period during which NAC was expected to exert its maximal beneficial effect, if any. An intravenous route for NAC would have required daily invasive treatment administration, and sedation of the animals, which did not seem acceptable; furthermore, oral and intravenous administrations have been reported to be equivalent [21]. Doses of NAC vary from 20 mg/kg to 400 mg/kg of body weight per day. Given that the bioavailability of orally administrated NAC is low (<10%), we decided to use high doses, closer to those used in acetaminophen poisoning than in chronic fibrotic diseases [22]. The pigs were put on a 60 mg/kg/day dose of NAC as part of a preliminary experiment and results showed improvement of the esophageal diameter at day 15 with no side effects. This propelled us to increase the NAC dose to 100 mg/kg/day. The use of higher doses, up to 400 mg/kg/day, has been reported, but is commonly associated with frequent side effects [23]. Such anaphylactoïd reactions, including vomiting, could have biased the evaluation of food intake, esophageal mucosa, and symptoms related to esophageal stricture. We did not record such anaphylactoïd reactions in the animals we treated. However, insufficient local concentration could also explain these results and could warrant another attempt of NAC administration, directly into esophageal wounds.

The second issue is the timing of the clinical and histological assessment. We chose the 21^st^ postoperative day as the day of endpoint assessment because it was consistent with the timing of stricture development in this model, generally observable between days 10 and 15 after ESD. Animals were kept in good condition after day 15, using iterative endoscopic dilations. Of note is the fact that these dilations might have played a role in the delayed reepithelialisation observed in some animals. It can be hypothesized that an earlier assessment of clinical and histological endpoints might have shown a more significantly different data, but the absence of any observable benefit at 21 days suggests such findings would not have been highly relevant to further clinical use of NAC.

A third hypothesis for NAC inefficiency is the severe and systematic nature of oesophageal strictures in pigs (possibly due to continuous acid aggression in conditions of stress). This makes our swine model particularly heuristic for the study of stricture development and prevention: if a treatment provides positive clinical results in this model, it should be all the more efficient in humans, but one would expect that NAC should at least exhibit some histological effect.

NAC has been found to have a direct antioxidant effect as well as to stimulate glutathione synthesis, a major endogenous intracellular antioxidant. It also plays a role in modulating the proinflammatory cytokine response [24], notably by inhibiting TGF-β1 [12] and abrogating the fibrogenic properties of pathological fibroblasts [14]. Post-endoscopic esophageal strictures are characterized by an intense local inflammatory reaction, which prevents reepithelialisation taking place and quickly makes way for cicatricial fibrosis. An increased level of reactive oxygen species (ROS) has been reported in esophageal ulcers, with the benefit of an antioxidant molecule (vitamin E) administration on esophageal wound healing [9]; along with these results, many stricture-preventing drugs, either antioxidant [25], antifibrotic [26-30], or anti-inflammatory [4,7,31], have been tested on esophageal wounds. The only treatment reported to be beneficial against esophageal strictures in human studies is local corticosteroid injection [4], although these results are contradictory, with previous animal studies reporting severe complications related to triamcinolone injection [7]. These data have led us to consider NAC for post-endoscopic esophageal stricture prevention. Despite an antioxidant effect suggested by the results of immunohistochemistry (although the difference between both signals on quantitative measurements did not reach statistical significance), NAC did not, in our work, prevent esophageal stricture.

Considering our results, it seems that several mechanisms could be at stake in cicatricial esophageal fibrosis. In particular, mucosal defect and resulting acid, and bacterial aggressions of the submucosa could play a role; therefore, stricture prevention strategies focusing on covering and protecting the injured esophagus should be developed, even if recent studies, including ones done on humans, either report insufficient efficacy [32], or show an improvement of esophageal wound healing only in partial mucosal defects [7,8,33-35], where strictures are uncommon. However, the use of growth factors, extracellular membranes, or bio-scaffolds or cellular therapy, makes their translation into daily practice unlikely for the present moment. This data confirm that preventive treatment should prevail against curative ones regarding post-endoscopic esophageal strictures.

## Conclusions

Our study failed to prove that an antioxidant such as NAC can improve the outcome of tissue repair in a model of severe post-ESD esophageal stricture, when used as a single agent. Its antioxidant activity did not prevent fibroinflammatory cicatricial esophageal stricture formation at the 21st postoperative day. Extensive esophageal endoscopic submucosal dissection might become an alternative choice to esophagectomy for Barrett’s esophagus with high-grade dysplasia or early adenocarcinoma; hence, further analysis of esophageal wound healing will be needed, in order to develop new stricture-preventing treatments.

## Methods

### Animal experiments

The experimental protocol received approval from the scientific committee of the Surgical School of Paris (Ecole de Chirurgie de l’ Assistance Publique des Hôpitaux de Paris, 7 rue du Fer à Moulin, 75005 Paris, France), and experiments were performed according to the standard guidelines of the French Ministry of Agriculture that regulates animal research in France.

Twelve 30 to 35 kg pigs, originating from the same farm, were used in the study. The pigs were accommodated at our facility 48 hours before the procedure took place. Endoscopies were performed under general anesthesia. All animals were prepared for anesthesia with a 12-hour diet and were administered an intramuscular injection of 10 mg/kg ketamine and 2 mg/kg azaperone, 30 minutes before induction. Following induction with 8 mg/kg intravenous 1% propofol and endotracheal intubation, anesthesia was maintained through inhalation of 1% to 2% isoflurane. All animals received an intravenous infusion of 10 mg/kg/h crystalloid solution.

### Endoscopic submucosal dissection procedure

Upper gastrointestinal endoscopies were performed using a standard gastroscope (Fujinon EG450D; Fujinon, Sataima, Japan), through an overtube. A senior endoscopist carried out and/or directly supervised the procedure at all times. Circumferential submucosal endoscopic dissection of the distal third of the esophagus was performed according to the previously reported technique [10]. Briefly, two circumferential incisions reaching the superficial submucosal layer were made following a submucosal injection of indigo carmine-stained saline; these two incisions defined a mucosal cylinder of 5 cm in the lower third of the esophagus, 5 cm above the gastroesophageal junction. The cylinder was removed by blunt submucosal dissection, using a mucosectomy cap (ST hood DH-19 GR; Fujinon, Sataima, Japan), which allowed for cleaving of the mucosa and superficial submucosa from the underlying submucosa by gently scraping the esophageal wall in a back and forth motion, from the proximal to the distal incision. The resected mucosal cylinder was then retrieved using the endoscope’s suction channel.

### Study design

Twelve pigs were randomly allocated to a treatment and a control group. The first group of six pigs was given 100 mg/kg/day of NAC (Mucomyst; Bristol-Myers Squibb, Princeton, NJ, USA) in drinking water for seven days before the procedure. After circumferential endoscopic submucosal dissection (CESD), the pigs received NAC treatment, at the same dose, until the 21^st^ postoperative day. A seven-day course of omeprazole 40 mg/day and broad-spectrum antibiotics was also given: 10 mg/kg extended-release benzathine benzylpenicillin G + penicillin procaine (Duphapen; Pfizer, NewYork, NY, USA) for the first four days; then 1 g/day amoxicillin (Clamoxyl®, GlaxoSmithKline, Brentford, UK) for three days. The control group (group 2) received proton-pump inhibitors and broad-spectrum antibiotics at the same doses.

The animals were given a liquid diet following each procedure, and were gradually introduced to a solid food diet. The pigs were kept in the Surgical School of Paris with daily examination and weekly veterinary follow-up and endoscopic examination. An endoscopic balloon dilation was performed if clinical symptoms suggested that an esophageal stricture had occurred. Investigators prospectively collected clinical data on a weekly basis, such as global clinical status, which included general behavior, food intake, and occurrence of regurgitations or vomiting. Investigators also recorded any endoscopic findings, width of esophageal lumen (evaluated with a biopsy forceps), and number of balloon dilations.

Twenty-eight days after the procedure, or earlier in case of major complication (esophageal perforation, death), animals were euthanized with 100 mg/kg intravenous injection of 7 mg pentobarbital (Dolethal; Vétoquinol, Paris, France), and underwent a necropsy with esophagectomy afterward.

### Histological analysis

Specimens were fixed in 10% buffered formalin, embedded in paraffin, and processed into 5 μm-thick sections, after gross morphological examination of the size, weight, and thickness of the esophageal wall. The specimens were then stained with hematoxylin-eosin-safran (HES), and Masson’s trichrome. Histological slides were digitized with the NDPI Nanozoomer (Hamamatsu Photonics, Hamamatsu City, Japan), and submitted to quantitative analysis of fibrosis, granulation tissue, and inflammatory cell infiltrate by a senior pathologist expert in digestive pathology. The following measurements were made on each digitized image of trichrome-stained slides: maximal thickness of fibrosis, from the muscularis propria to the epithelium, and maximal thickness of the granulation tissue, from the esophageal lumen to the submucosal fibrosis. Furthermore, the inflammatory cell infiltrate was characterized as being acute (predominance of polynuclear cells, high cell density) or chronic (predominance of lymphocytes or plasmocytes, low cell density).

Immunohistochemistry on paraffin sections was performed with monoclonal antibodies to 8-hydroxy-2’ deoxyguanosine (8-OH DG) (ab26842; Abcam, Cambridge, UK) and goat polyclonal secondary antibodies to mouse IgG1 (ab 97240; Abcam, Cambridge, UK), in order to assess the intensity of oxidative stress in the esophageal wall. The intensity of the signal was analyzed using the ImageJ software, the highest numbers corresponding to the lowest signal.

### Statistical analysis

Statistical analysis was performed using GraphPad Prism (GraphPad Software, La Jolla, CA, USA). Continuous data is expressed as median values and range, and compared with a nonparametric Mann–Whitney test. Categorical data is expressed in percentages and compared with a Fisher’s exact test.

### End points

The primary end point was the diameter of the esophagus measured via endoscopy on day 21 following the operation.

The secondary end points were: global clinical evaluation of the pigs on day 21, number of endoscopic dilations required, thickness of the granulation tissue and the fibrotic layer in the esophageal wall, and antioxidant effect of NAC, as measured by 8-OH DG staining.

## Abbreviations

CESD: Circumferential endoscopic submucosal dissection; HES: Hematoxylin-eosin-safran; IL: Interleukin; NAC: N-acetylcysteine; NS: Not significant; ROS: Reactive oxygen species; 8-OH DG: 8-hydroxy-2’ deoxyguanosine; TGF: Transforming growth factor; TNF-α: Tumor necrosis factor-alpha.

## Competing interests

The authors declare that they have no competing interests, and do not report any funding source.

## Authors’ contributions

MB carried out the endoscopies and histologic study, the statistical analysis and drafted the manuscript. F. Batteux participated in the design of the study. F. Beuvon supervised all histological evaluations. LM and AC supervised all endoscopic procedures. CP helped carry out the endoscopies and the histologic study. SC participated in the design of the study and corrected the draft. FP conceived of the study, and participated in its design and coordination and helped to draft the manuscript. All authors read and approved the final manuscript.
